# Comparison of heart deformation analysis and cine DENSE in volunteers and heart failure patients

**DOI:** 10.1186/1532-429X-18-S1-P45

**Published:** 2016-01-27

**Authors:** Daniel A Auger, Claire Stump, Marie-Pierre Jolly, Andreas Greiser, Kenneth C Bilchick, Frederick H Epstein

**Affiliations:** 1 grid.27755.32000000009136933XBiomedical Engineering, University of Virginia, Charlottesville, VA USA; 2 grid.27755.32000000009136933XMedicine, Cardiovascular Medicine, University of Virginia, Charlottesville, VA USA; 3 grid.415886.6Medical Imaging Technologies, Siemens Healthcare, Princeton, NJ USA; 4 Imaging & Therapy Magnetic Resonance, Siemens Healthcare, Nürnberg Area Germany; 5 grid.27755.32000000009136933XRadiology and Medical Imaging, University of Virginia, Charlottesville, VA USA

## Background

Post-processing methods have been developed to quantify myocardial strain from steady-state free-precession (SSFP) cine images. In the left ventricle (LV), global strain measurements from SSFP in healthy volunteers^1,2^ and in some types of heart disease compare favorably with myocardial tagging as a reference. We compared heart deformation analysis (HDA) of SSFP images and cine Displacement Encoding with Stimulated Echoes (DENSE) for the assessment of global and segmental circumferential strain (Ecc) and derivative indices in healthy volunteers and heart failure (HF) patients with left bundle branch block (LBBB).

## Methods

Imaging was performed on a 1.5T Siemens Avanto MR system in 3 short-axis planes in 6 volunteers and 29 patients with HF-LBBB. Ecc was computed from SSFP images using HDA (Trufi Strain, Siemens Healthcare, Erlangen) and from cine DENSE using previously described methods^3,4^. The mean and standard deviation of global Ecc were compared and the correlation between HDA and cine DENSE was assessed. The circumferential uniformity ratio estimate computed with singular value decomposition (CURE-SVD) is a quantitative index of mechanical dyssynchrony based on segmental Ecc, where values near 1 indicate synchrony and lower values indicate dyssynchrony^5^. CURE-SVD was computed for all subjects using HDA and DENSE

## Results

**Figure 1:**
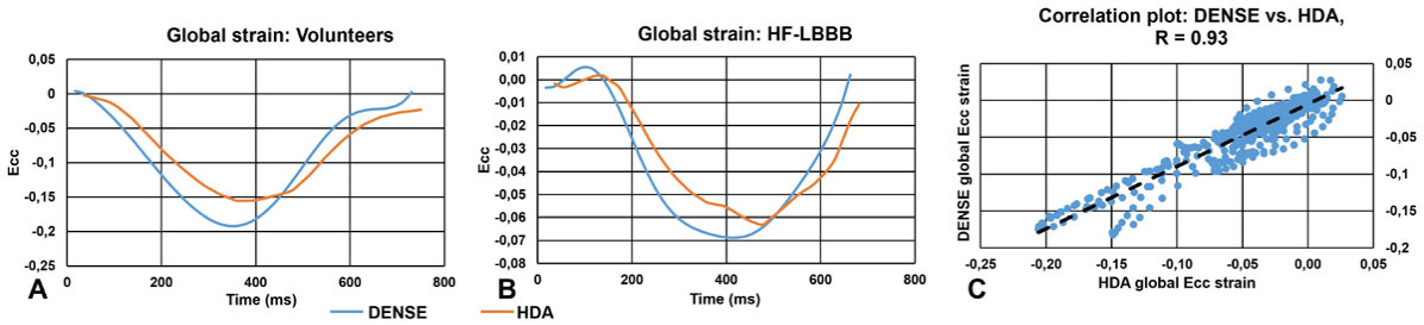
**Data shows good agreement between cine DENSE and HDA for global Ecc**. (A, B) Cine DENSE and HDA global Ecc curve for healthy volunteer and patient with HF-LBBB. (C) DENSE vs. HDA global strain correlation plot for all data.

**Figure 2:**
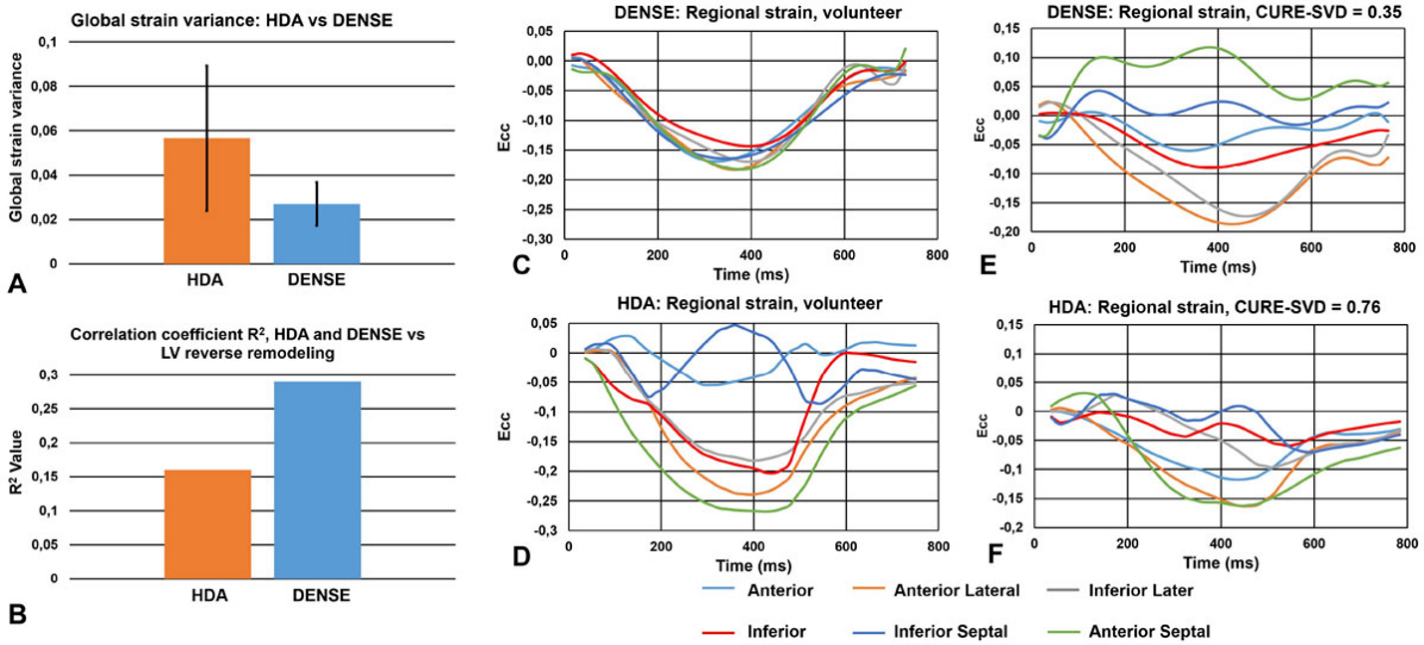
**Data shows Ecc segmental variation in HDA compared to cine DENSE**. (A) Mean ± standard deviation of segmental strain variation across all slices. (B) Correlation coefficient (R^2^) of linear plot for both HDA and DENSE vs LV reverse remodeling. (C, D) Cine DENSE and HDA regional Ecc curves for a healthy volunteer. (E, F) Cine DENSE and HDA regional Ecc curves for a patient with HF-LBBB and corresponding CURE-SVD values.

Fig. 1A-B show examples of good agreement of global Ecc between HDA and DENSE for a volunteer and patient, and Fig. 1C shows a good correlation of global Ecc for all subjects. For the segmental analysis, we found greater variability in Ecc (Fig 2A) as assessed by HDA compared to cine DENSE in volunteers (p = 0.001), which in some cases mimicked segmental dysfunction, Fig 2B-C. CURE-SVD as assessed using both DENSE and HDA discriminated patients from volunteers. CURE-SVD for volunteers was higher with DENSE than HDA (more synchronous) (0.91 ± 0.0003 vs 0.82 ± 0.02, p < 0.05) and in patients was lower with DENSE than HDA (more dyssynchronous) (0.45 ± 0.03 vs 0.52 ± 0.02, p < 0.05). An example is shown in Fig 2E-F. Using a linear regression model with CURE-SVD as the predictor and percent change in LV end systolic volume 6 months after CRT as the outcome, DENSE had a better correlation (R^2^ = 0.29, p = 0.002) than HDA (R^2^ = 0.16, p = 0.03), Fig. 2A.

## Conclusions

HDA and cine DENSE show good agreement for global Ecc. For regional Ecc, HDA shows greater variability including apparent false-positive segmental dysfunction in some volunteers. While HDA measurements of dyssynchrony can discriminate HF-LBBB from healthy volunteers (p < 0.01), it underestimates the degree of synchrony in volunteers and the degree of dyssynchrony in patients compared to cine DENSE. CURE-SVD by cine DENSE has a better correlation to LV remodeling compared to HDA.
